# Palbociclib Combined with an Aromatase Inhibitor in Patients with Breast Cancer with Lung or Liver Metastases in US Clinical Practice

**DOI:** 10.3390/cancers15215268

**Published:** 2023-11-02

**Authors:** Adam Brufsky, Xianchen Liu, Benjamin Li, Lynn McRoy, Connie Chen, Rachel M. Layman, Hope S. Rugo

**Affiliations:** 1 UPMC Hillman Cancer Center, Department of Medicine, Division of Hematology/Oncology, University of Pittsburgh Medical Center, Pittsburgh, PA 15213, USA; 2 Pfizer Inc., New York, NY 10001, USA; jasonxc.liu@pfizer.com (X.L.); benjamin.li@pfizer.com (B.L.); lynn.mcroy@pfizer.com (L.M.); connie.chen@pfizer.com (C.C.); 3 Department of Breast Medical Oncology, The University of Texas MD Anderson Cancer Center, Houston, TX 77030, USA; rlayman@mdanderson.org; 4 Department of Medicine (Hematology/Oncology), University of California San Francisco Helen Diller Family Comprehensive Cancer Center, San Francisco, CA 94158, USA; hope.rugo@ucsf.edu

**Keywords:** metastatic breast cancer, palbociclib, liver metastasis, lung metastasis, visceral metastases

## Abstract

**Simple Summary:**

Palbociclib combined with an aromatase inhibitor (AI) has been shown to be effective in clinical trials for people with HR+/HER2− breast cancer that has spread to other areas of the body, such as the lungs or liver. Evidence of palbociclib effectiveness in routine clinical practice can provide complementary support for clinical trial findings. This study used electronic health records of people with breast cancer that had spread to their lungs and/or liver to determine how well palbociclib plus an AI worked compared to an AI alone. The study showed that palbociclib plus an AI compared with an AI alone was associated with a 38% or 27% reduction in the risk of death for patients with breast cancer that had spread to the lungs or liver, respectively. These findings support the use of palbociclib plus an AI for people whose breast cancer has spread to their lungs or liver.

**Abstract:**

A cyclin-dependent kinase 4/6 inhibitor combined with endocrine therapy is the standard of care for patients with hormone receptor-positive/human epidermal growth factor 2-negative (HR+/HER2−) metastatic breast cancer (mBC), but real-world effectiveness data for patients with lung or liver metastases are limited. This retrospective study included data from the US Flatiron Health database of patients with HR+/HER2− mBC and lung or liver metastases treated with first-line palbociclib (PAL) plus an aromatase inhibitor (AI) or an AI alone in routine clinical practice. Overall survival (OS) and real-world progression-free survival (rwPFS) were assessed. A total of 891 patients were included (622 with lung metastasis, 376 with liver metastasis, and 107 with both lung and liver metastasis). After stabilized inverse probability of treatment weighting to balance patient characteristics, PAL + AI versus AI alone was associated with significantly prolonged OS (HR = 0.62; *p* < 0.001) and rwPFS (HR = 0.55; *p* < 0.001) in patients with lung metastases and numerically longer OS (HR = 0.73; *p* = 0.056) and significantly longer rwPFS (HR = 0.57, *p* < 0.001) for those with liver metastases. Overall, PAL + AI versus AI alone was associated with prolonged OS and rwPFS in routine clinical practice, supporting the use of first-line PAL + AI for patients with HR+/HER2− mBC with lung and/or liver metastases.

## 1. Introduction

In 2018, more than 140,000 women were estimated to be living with metastatic breast cancer (mBC), a population expected to grow to more than 169,000 by 2025 [1]. Despite recent advances in treatment, the 5-year survival rate for patients with mBC remains low (31%), and it is estimated that more than 43,000 women in the United States (US) will die from breast cancer in 2023 [2]. Approximately 70% of all breast cancers are hormone receptor-positive (HR+)/human epidermal growth factor receptor 2-negative (HER2−) breast cancer [2], which most frequently metastasizes to bone (59%), lung (22%), and liver (15%) [3]. Visceral (e.g., lung and liver) metastases have been associated with poorer outcomes for patients with mBC than for those with non-visceral (e.g., bone, skin, and lymph nodes) spread [4,5,6]. Retrospective analyses from the Surveillance, Epidemiology, and End Results (SEER) database observed a median overall survival (OS) of 16 months (*n* = 2940) [7] for patients with HR+/HER2– mBC and lung metastases and 21 months for liver metastases (*n* = 1276) [8], compared with 43 months for patients with bone-only metastases (*n* = 4096) [9]. Patients with visceral spread receiving endocrine therapy (ET) plus placebo in phase 3 clinical trials have shorter progression-free survival (PFS) and OS than those without visceral spread. One study reported a median PFS of 12.3 versus 17.0 months for patients with versus without visceral disease [10], while another reported a median PFS of 7.2 versus 15.4 months for patients with versus without liver metastases [11]. The median OS was also shorter for patients with versus without liver involvement (38.1 vs. 56.9 months) [12]. Collectively, the current data indicate that patients with mBC that has spread to the lungs or liver have an unfavorable prognosis and that liver involvement, in particular, may present considerable treatment challenges [13,14,15]. As such, there is an urgent unmet need for therapies that are effective for these patient populations.

A cyclin-dependent kinase 4/6 (CDK4/6) inhibitor in combination with an aromatase inhibitor (AI) is a standard first-line treatment for patients with HR+/HER2− mBC [16,17]. Palbociclib, the first-in-class CDK4/6 inhibitor, has been approved for use in the US for the treatment of estrogen-receptor positive (ER+)/HER2− mBC since 2015 [18]. A subgroup analysis of patients with visceral disease from the phase 3 PALOMA-2 trial demonstrated a significant PFS benefit for those in the palbociclib plus letrozole arm (19.3 months) compared with patients in the placebo plus letrozole arm (12.3 months; hazard ratio [HR] = 0.62, 95% CI: 0.47–0.81; *p* < 0.0005) [10] and a numerically prolonged median OS (48.1 vs. 44.8 months) [19]. In the 8 years since it was first approved, more than 173,000 patients in the US and more than 665,000 patients worldwide have been prescribed palbociclib in routine clinical practice. As a result, sufficient real-world data are now available to assess palbociclib effectiveness in key subgroups of interest. Real-world data in breast cancer research complement randomized controlled trials by offering insights into patient characteristics, treatment patterns, and outcomes in routine clinical practice [20,21].

Although real-world evidence supporting the use of CDK4/6 inhibitors, particularly palbociclib, as first-line therapy in patients with HR+/HER2− mBC has grown [21,22,23,24,25,26,27,28,29,30,31,32], comparative effectiveness data are limited for CDK4/6 inhibitors plus an AI versus an AI alone, including for patients with visceral metastases [33,34]. Two previous real-world data studies using the Flatiron database have examined the effectiveness of palbociclib plus ET in patients with HR+/HER2− mBC and visceral metastases. One study by Rugo et al. [27] found significantly longer OS and real-world (rw)PFS for patients with visceral metastasis (*n* = 848) treated with palbociclib plus an AI versus an AI alone. However, the subgroup analysis was univariate and did not separately assess outcomes for patients with lung or liver metastasis. Another study (*n* = 551) demonstrated that palbociclib plus letrozole was associated with significantly prolonged rwPFS (HR = 0.56) and OS (HR = 0.58) for patients with HR+/HER2− mBC with visceral metastasis (lung or liver) compared with letrozole alone [34]. An examination of the individual subgroups of lung and of liver metastatic sites showed a significant rwPFS and OS benefit of palbociclib plus letrozole versus letrozole alone for patients with lung metastases and a significant OS benefit for patients with liver metastases, but the study had a limited subgroup sample size. Here we present the results of an expanded analysis of the Rugo et al. study [27], in which we examined the comparative effectiveness of first-line palbociclib plus an AI versus an AI alone in pre- and post-menopausal women and men with HR+/HER2− mBC who have lung and/or liver metastases treated in US routine clinical practice.

## 2. Methods

### 2.1. Study Design and Data Source

This retrospective study used electronic health records from the Flatiron Health longitudinal database, which contains de-identified patient data from >280 cancer clinics, representing more than 3.5 million patients with cancer being actively treated in the US. This database has undergone extensive validation and has been used for multiple real-world studies of patients with breast cancer [27,34,35,36].

### 2.2. Patients

This study included women and men (≥18 years of age) who were diagnosed with HR+/HER2− mBC and visceral disease and who initiated palbociclib plus an AI or an AI alone as a first-line treatment between February 2015 and March 2020 in routine clinical practice, with a data cut-off date of September 2020. Visceral disease was defined as metastatic disease in the lung and/or liver. Patient follow-up was conducted from the start of therapy to the data cut-off date, death, last visit, or the date of initiation of the next line of therapy for patients with two or more lines of therapy, whichever came first, with a potential minimum follow-up of 6 months from the index date until the data cut-off date. The exclusion criteria were evidence of any prior treatments with CDK4/6 inhibitors, endocrine treatments, or chemotherapy in the mBC setting. Patients with first structured activity >90 days from the mBC diagnosis date or with missing relevant unstructured documents in the Flatiron database were also excluded from the study.

### 2.3. Outcomes

The primary outcome of this study was OS, defined as the number of months from the start of palbociclib plus an AI or an AI alone until death due to any cause [27]. A previously validated composite mortality dataset benchmarked against the National Death Index was used to determine date of death [37,38]. A secondary outcome was the rwPFS, defined as the number of months from the start of treatment with palbociclib plus an AI or an AI alone to the date of the first documentation of real-world progressive disease or death due to any cause, whichever occurred first [26]. Disease progression was evaluated by the treating clinician based on radiology, tissue biopsy, laboratory evidence, or clinical assessment. If patients did not die or experience disease progression, those who received two or more lines of treatment were censored at the date of initiation of the next line of treatment, and those who received one line were censored at their last visit date during the study period.

### 2.4. Statistical Analysis

Descriptive statistics were used for baseline demographic and clinical characteristics. Treatment comparative analyses were conducted for patients with liver, lung, and both liver and/or lung metastases. Three methods were used for comparative analysis: (1) an unadjusted analysis without controlling for baseline patient characteristics, (2) a stabilized inverse probability of treatment weighting (sIPTW) method (primary analysis) to balance baseline demographic and clinical characteristics between the treatment groups and control for confounding variables, and (3) a 1:1 propensity score matching (PSM) method as a sensitivity analysis to assess the robustness of the sIPTW results. Both the sIPTW and PSM are based on propensity scores calculated by a multivariable binomial logistic regression model. Variables included in the model were age group, sex, race/ethnicity, practice type, disease stage at initial diagnosis, Eastern Cooperative Oncology Group (ECOG) performance status score, bone disease, visceral disease, interval from initial breast cancer diagnosis to mBC diagnosis, and number of metastatic sites.

The weighted Kaplan–Meier method was used to summarize and display median survival times and 95% CIs for OS and rwPFS endpoints. The weighted Cox proportional hazards model was used to compute the HRs and corresponding 95% CIs. All analyses were performed using SAS version 9.1.4 or higher (SAS Institute, Cary, NC, USA).

## 3. Results

### 3.1. Patients

Data from 891 patients with HR+/HER2− mBC and lung and/or liver metastases were included in the study. Of these patients, 622 (69.8%) had lung metastasis, 376 (42.2%) had liver metastasis, and 107 (12.0%) had both lung and liver metastases. Overall, 480 patients received palbociclib plus an AI, and 411 received an AI alone. For patients with lung metastases, 326 (52.4%) received palbociclib plus and AI, whereas 296 (47.6%) received an AI alone. For patients with liver metastases, 211 (56.1%) were given palbociclib plus an AI, whereas 165 (43.9%) were given an AI alone.

Patient demographic and clinical characteristics are reported by the site of metastasis (Table 1 and Table 2) and for the overall cohort of patients with lung and/or liver metastasis (Supplementary Table S1) for the unadjusted, sIPTW, and PSM analyses. The median age of patients treated with palbociclib plus an AI was 66 years, and 71 years for those treated with an AI alone. Patients in the palbociclib plus AI group were more likely to be White, have an ECOG performance status score of 0, and have more than three sites of metastases. However, patient characteristics in both treatment groups were generally well balanced after sIPTW and PSM in the overall cohort and in the lung and liver metastasis subgroups, as demonstrated by the standardized difference being <0.1. The median follow-up duration (interquartile range) was 24.5 (23.7) and 20.5 (28.2) months for patients treated with palbociclib plus an AI and those treated with an AI alone, respectively.

### 3.2. Overall Survival

**Figure 1:**
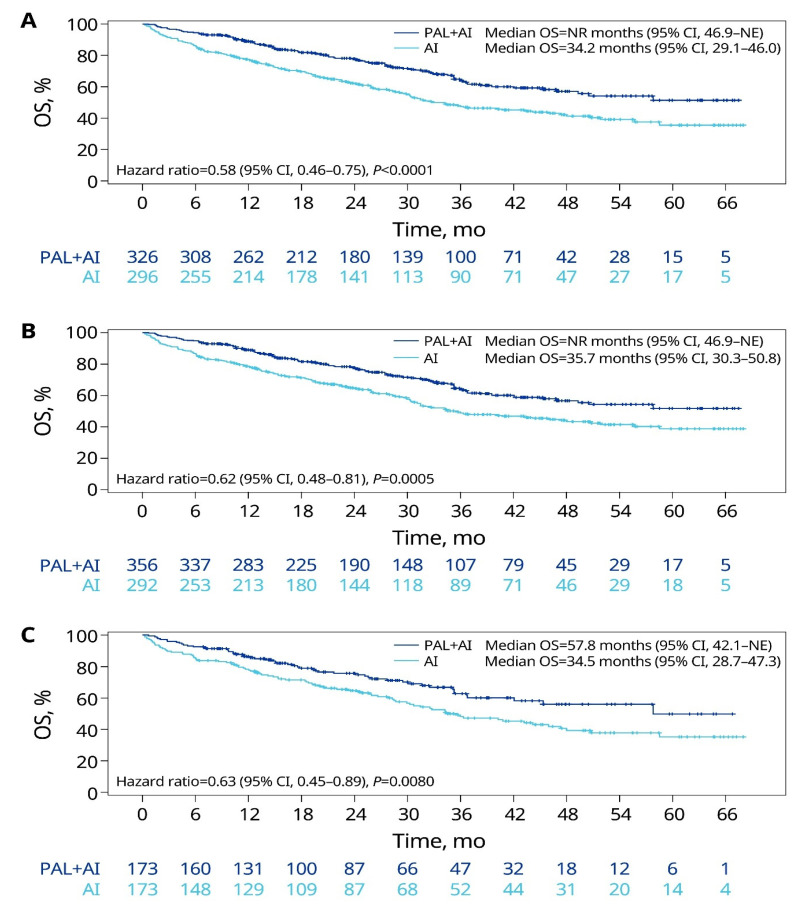
OS of patients with lung metastasis in the unadjusted (**A**), sIPTW (**B**), and PSM (**C**) analyses. AI, aromatase inhibitor; CI, confidence interval; NE, not estimable; NR, not reached; OS, overall survival; PAL, palbociclib; PSM, propensity score matching; sIPTW, stabilized inverse probability of treatment weighting.

In the unadjusted analysis, patients with lung metastasis treated with palbociclib plus an AI had a significantly longer median OS than those treated with an AI alone (not reached [NR] vs. 34.2 months; HR = 0.58; *p* < 0.0001; Figure 1A). After sIPTW (primary analysis), palbociclib plus AI was associated with a significantly longer median OS compared with an AI alone (NR [95% CI: 46.9–NE] vs. 35.7 months [95% CI: 30.3–50.8]; HR = 0.62, 95% CI: 0.48–0.81; *p* = 0.0005; Figure 1B). The PSM sensitivity analysis of the median OS supports the results of the primary analysis (Figure 1C).

**Figure 2:**
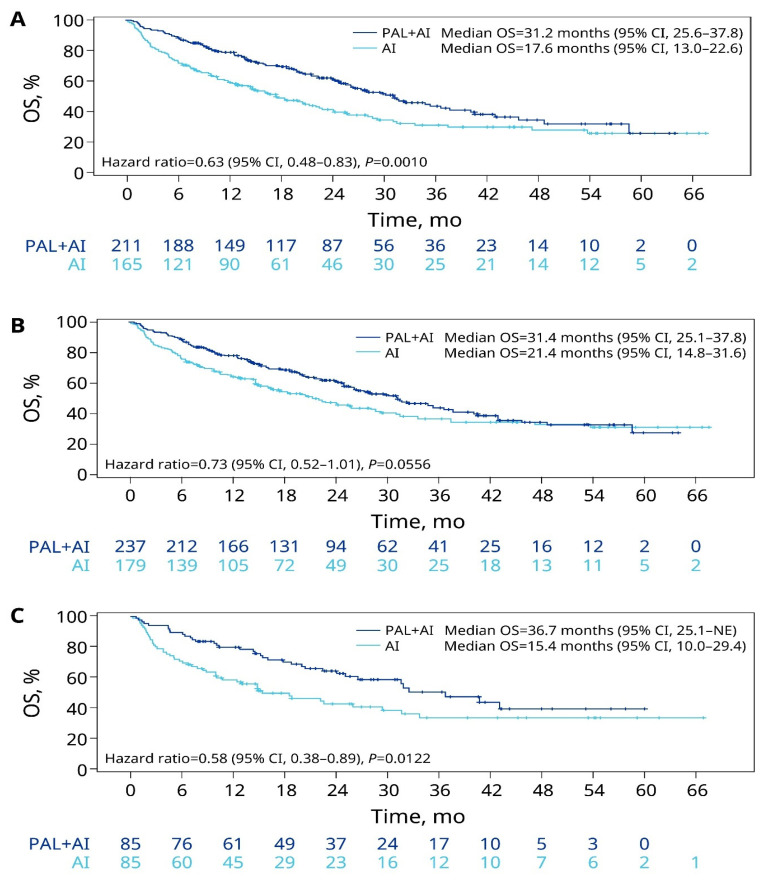
OS of patients with liver metastasis in the unadjusted (**A**), sIPTW (**B**), and PSM (**C**) analyses. AI, aromatase inhibitor; CI, confidence interval; NE, not estimable; OS, overall survival; PAL, palbociclib; PSM, propensity score matching; sIPTW, stabilized inverse probability of treatment weighting.

In the unadjusted analysis for patients with liver metastasis, the median OS was significantly prolonged for patients receiving palbociclib plus an AI versus an AI alone (31.2 vs. 17.6 months; HR = 0.63, *p* = 0.001; Figure 2A). After sIPTW, the median OS was 31.4 months (95% CI: 25.1–37.8) for the palbociclib plus AI group compared with 21.4 months (95% CI: 14.8–31.6) for the AI-alone group, but the difference did not reach statistical significance (HR = 0.73, 95% CI: 0.52–1.01; *p* = 0.0556; Figure 2B). However, after PSM, the median OS was significantly prolonged for the palbociclib plus AI group (36.7 months) compared with the AI-alone group (15.4 months; HR = 0.58; *p* = 0.0122; Figure 2C).

For the cohort of patients with lung and/or liver metastases, treatment with palbociclib plus an AI was associated with a significantly prolonged median OS in the unadjusted, sIPTW, and PSM analyses compared with treatment with an AI alone (Supplementary Figure S1). After sIPTW, the median OS was significantly extended in the palbociclib plus AI group compared with the AI-alone group (49.3 months [95% CI: 39.5–NE] vs. 31.5 months [95% CI: 28.5–36.8]; HR = 0.64, 95% CI: 0.52–0.79; *p* < 0.0001).

### 3.3. Real-World Progression-Free Survival

**Figure 3:**
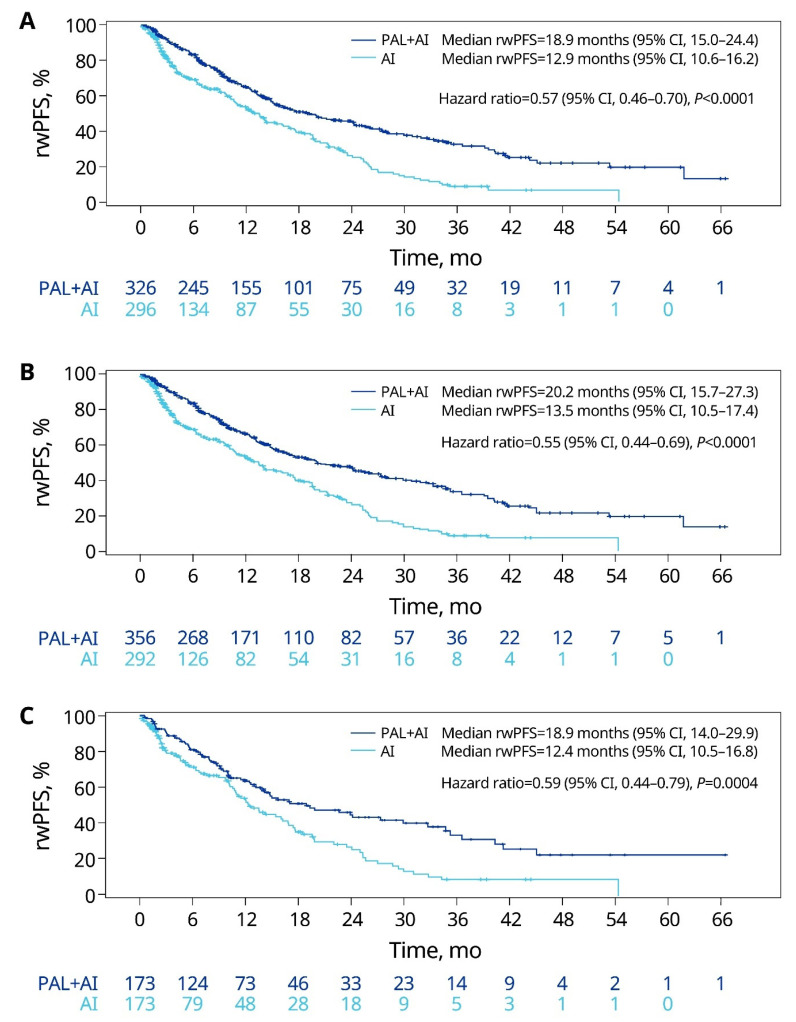
rwPFS of patients with lung metastasis in the unadjusted (**A**), sIPTW (**B**), and PSM (**C**) analyses. AI, aromatase inhibitor; CI, confidence interval; PAL, palbociclib; PSM, propensity score matching; rwPFS, real-world progression-free survival; sIPTW, stabilized inverse probability of treatment weighting.

In the unadjusted analysis of patients with lung metastasis, palbociclib plus AI was associated with a significantly prolonged median rwPFS compared with an AI alone (18.9 months vs. 12.9 months; HR = 0.57; *p* < 0.0001; Figure 3A). After sIPTW, the median rwPFS was significantly longer in the palbociclib plus AI group compared with the AI-alone group (20.2 months [95% CI: 15.7–27.3] vs. 13.5 months [95% CI: 10.5–17.4]; HR = 0.55, 95% CI: 0.44–0.69; *p* < 0.0001; Figure 3B). The results of the PSM analysis were consistent with those of the sIPTW analysis (Figure 3C).

**Figure 4:**
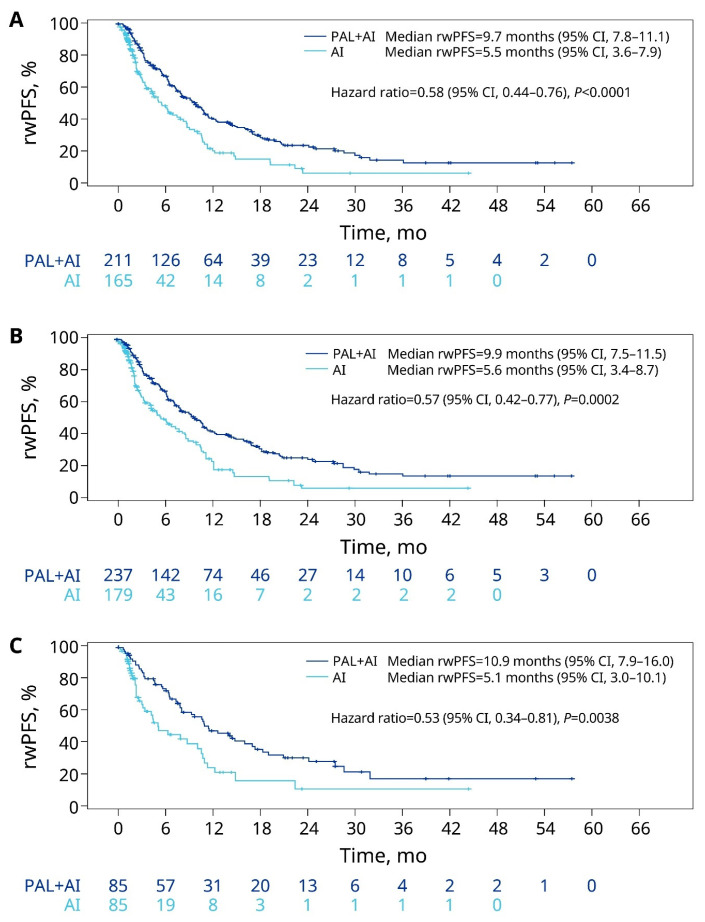
rwPFS of patients with liver metastasis in the unadjusted (**A**), sIPTW (**B**), and PSM (**C**) analyses. AI, aromatase inhibitor; CI, confidence interval; PAL, palbociclib; PSM, propensity score matching; rwPFS, real-world progression-free survival; sIPTW, stabilized inverse probability of treatment weighting.

For patients with liver metastasis, the rwPFS was significantly prolonged with palbociclib plus an AI (9.7 months) versus an AI alone (5.5 months; HR = 0.58; *p* < 0.0001; Figure 4A) in the unadjusted analysis. After sIPTW, treatment with palbociclib plus AI was associated with a significantly longer rwPFS compared with an AI alone (9.9 months [95% CI: 7.5–11.5] vs. 5.6 months [95% CI: 3.4–8.7]; HR = 0.57, 95% CI: 0.42–0.77; *p* = 0.0002; Figure 4B). The PSM analysis also showed a significantly longer rwPFS in the palbociclib plus AI group versus the AI-alone group (Figure 4C).

In the overall cohort of patients with lung and/or liver metastases, the unadjusted, sIPTW, and PSM analyses each revealed a significantly longer rwPFS for patients receiving palbociclib plus AI compared with an AI alone (Figure S2). After sIPTW, rwPFS was significantly prolonged in the palbociclib plus AI group versus the AI-alone group (17.0 months [95% CI: 14.2–20.0] vs. 10.7 months [95% CI: 8.9–12.9]; HR = 0.57; 95% CI: 0.48–0.69; *p* < 0.0001).

## 4. Discussion

Lung and liver are the second and third most common sites of breast cancer metastasis and have generally unfavorable prognoses [3,4]. In this study, we assessed the comparative effectiveness of palbociclib plus an AI versus an AI alone for survival outcomes among patients with HR+/HER2− mBC that had metastasized to the lungs or liver. The addition of palbociclib to an AI was associated with clinically meaningful benefits in the OS and rwPFS of patients with lung and/or liver metastases. Although the median OS was only numerically longer for patients with liver metastases, the observed ≥ 10-month extension is clinically meaningful and a tangible benefit for patients. While caution should be exercised when making comparisons between real-world data studies and randomized controlled trials, we note that our findings are broadly consistent with the visceral metastases subgroup analysis from the PALOMA-2 trial, which reported clinical benefits in the primary endpoint of PFS for palbociclib plus letrozole over placebo plus letrozole [10,19]. Furthermore, our study confirms and extends previous real-world findings [34] by providing additional support to the effectiveness of palbociclib plus an AI for patients with mBC and visceral involvement.

Studies drawing from data preceding the advent of modern therapies, such as CDK4/6 inhibitors, reported survival outcomes ranging from 16 to 28 months for mBC that spreads to lung or liver [7,8,39]. However, accruing evidence suggests that there are different outcomes for the spread of malignant tumors to different organs and that visceral metastasis may comprise multiple, discrete conditions. A retrospective study based on data collected from 2010 to 2015 found that the OS in patients with mBC and lung metastases did not differ significantly from those with bone metastases (HR = 0.99, 95% CI: 0.90–1.10; *p* = 0.902), whereas those with liver metastases had significantly shorter OS (HR = 1.43, 95% CI: 1.27–1.60; *p* < 0.001) [15]. Similarly, a more recent real-world study reported a longer OS for patients with mBC and lung involvement than those with liver involvement who were treated with letrozole alone (40.3 months and 16.8 months, respectively) or palbociclib plus letrozole (NR and 30.1 months, respectively) [34]. A retrospective study of data collected in China from 2000–2019 observed that the risk of death more than doubled for patients with mBC and synchronous lung metastases who also had concomitant liver involvement (HR = 2.19, 95% CI: 1.70–2.82; *p* < 0.001) [40]. Although our study was not designed to compare outcomes between patients with lung and liver involvement, our results are consistent with these previous reports in reporting a longer OS for patients with lung metastases relative to those with liver metastases.

Many factors contribute to the clinical outcomes of patients with mBC, including their demographics, clinicopathologic features, tumor molecular subtype, treatment selection, and sequencing [15,41]. Meta-analyses of the data from clinical trials conducted from 1995–2014 demonstrated that patients with HR+ mBC with visceral non-liver metastases receiving ET alone were more likely to have a longer PFS (7.8 months), OS (32.8 months), and duration of clinical benefit (17.1 months) than those with liver metastases (3.9 months, 20.4 months, and 13.1 months, respectively) [6]. In another retrospective study assessing PFS in patients with HR+ mBC and lung (*n* = 138) or liver (*n* = 51) involvement treated with fulvestrant, the median PFS was 9.6 months and 3.9 months, respectively [42]. Importantly, our findings demonstrate that palbociclib plus an AI is associated with clinically meaningful reductions in the risk of death of 38% for patients with lung involvement and 27% for those with liver involvement. Furthermore, treatment with palbociclib plus an AI was associated with a 45% and 43% reduction in the risk of disease progression for patients with lung and liver metastases, respectively.

In this study, analysis of the overall cohort of patients with lung and/or liver involvement demonstrated superior OS and rwPFS outcomes for patients treated with palbociclib and AI compared with AI monotherapy. These results are consistent with meta- and pooled analyses of clinical trials of CDK4/6 inhibitor plus ET, which demonstrated a survival benefit in both OS (*n* = 1390; HR = 0.76; *p* < 0.001) [43] and PFS (*n* = 2094; HR = 0.58, 95% CI: 0.52–0.65) [44] for patients with mBC and visceral metastases compared with ET alone. It is possible that the site that comprises the largest proportion of patients who are in the broad visceral metastasis cohort is driving the effects observed in some studies. Given that the prevalence of lung metastases is greater than liver metastases in the overall population of patients with mBC, the treatment efficacy/effectiveness may be biased by patients with lung involvement. A related complication to the interpretation of outcomes for patients with mBC is concurrent spread to other visceral and non-visceral sites. In a previous analysis using the Flatiron Health database, approximately 40% of patients had bone-only metastasis. That study reported significant OS and PFS benefits for patients with bone-only metastases when receiving palbociclib plus an AI compared with an AI alone [27]. However, the current study is unable to shed light on the effects of concurrent spread to bone on the survival outcomes of patients with visceral metastases. Taken together, our findings support conducting separate analyses of patients with mBC with lung-only or liver-only spread in future studies, if the sample size is sufficient.

The current study has several limitations. As an observational study, only associations can be inferred between treatments and outcomes, but not causality. Retrospective database analyses may have incomplete or missing data and inaccurate data capture. Unlike clinical trials, disease progression was not assessed on a schedule and was not based on the Response Evaluation Criteria in Solid Tumors; therefore, rwPFS data were dependent on each oncologist’s interpretation of diagnostic scans and pathology reports. Treatments were not randomly assigned but were selected for each patient based on the treating physician’s judgment, resulting in potential treatment selection bias. Although sIPTW and PSM were used to balance patient characteristics, the effects of potential unmeasured confounders could not be adjusted for in these analyses. Finally, our results may not be generalizable outside of the US-based Flatiron Health network. It should be noted that our study did not separately analyze patients that had mBC spread to both lung and liver due to the small number of patients in this group. Future inquiries could focus on this population because these patients may exhibit worse outcomes than those with single-organ metastasis [41]. Separate analysis of patients with both lung and liver involvement would also permit independent analyses of patients with lung-only and liver-only metastases if the sample size is sufficient. Despite these limitations, the strengths of the study should be mentioned; these include a relatively large patient population that affords the statistical power necessary to permit separate comparative analyses of lung and liver subgroups with patient characteristics balanced by sIPTW, a database with validated death dates, and a long follow-up of up to 68 months.

## 5. Conclusions

Treatment with palbociclib plus an AI versus an AI alone in routine clinical practice was associated with OS and rwPFS benefits for patients with HR+/HER2− mBC with lung or liver metastasis. This finding is important given that visceral involvement is common in HR+/HER2− mBC, can be difficult to treat, and is associated with a poor prognosis. These data support the use of first-line palbociclib plus an AI for the treatment of patients with HR+/HER2− mBC with lung or liver metastases.

## Figures and Tables

**Table 1:** Baseline and clinical characteristics of patients with lung metastasis.

Characteristic	Unadjusted			sIPTW			PSM		
	Palbociclib + AI (*n* = 326)	AI Alone (*n* = 296)	Std Diff	Palbociclib + AI (*n* = 356)	AI Alone (*n* = 292)	Std Diff	Palbociclib + AI (*n* = 173)	AI Alone (*n* = 173)	Std Diff
Age at mBC diagnosis, years ^a^									
Mean (SD)	67.0 (10.5)	71.0 (9.8)	−0.3941	68.6 (11.1)	69.1 (10.1)	−0.0521	68.6 (10.3)	69.9 (9.4)	−0.1408
Median (IQR)	68 (15)	72 (16)		69 (15)	70 (16)		69 (16)	70 (15)	
Female sex ^a^ *n* (%)	323 (99.1)	290 (98.0)	−0.0919	348 (97.9)	288 (98.4)	0.0378	172 (99.4)	172 (99.4)	0.0000
Race ^a^ *n* (%)									
White	218 (66.9)	183 (61.8)	0.1055	234 (65.9)	192 (65.6)	0.0050	113 (65.3)	117 (67.6)	−0.0490
Black	26 (8.0)	28 (9.5)	−0.0526	30 (8.3)	24 (8.3)	0.0012	14 (8.1)	14 (8.1)	0.0000
Other	82 (25.2)	85 (28.7)	−0.0804	92 (25.8)	76 (26.1)	−0.0062	46 (26.6)	42 (24.3)	0.0531
Practice type									
Community	302 (92.6)	271 (91.6)	0.0402	327 (92.1)	269 (91.9)	0.0047	158 (91.3)	157 (90.8)	0.0202
Academic	24 (7.4)	25 (8.4)		28 (7.9)	24 (8.1)		15 (8.7)	16 (9.2)	
mBC disease stage at initial diagnosis, *n* (%)									
I	40 (12.3)	37 (12.5)	−0.0070	45 (12.7)	38 (12.9)	−0.0084	19 (11.0)	23 (13.3)	−0.0708
II	87 (26.7)	70 (23.6)	0.0701	91 (25.5)	76 (25.9)	−0.0072	50 (28.9)	44 (25.4)	0.0780
III	36 (11.0)	50 (16.9)	−0.1693	49 (13.8)	41 (13.9)	−0.0051	25 (14.5)	18 (10.4)	0.1229
IV	131 (40.2)	101 (34.1)	0.1257	130 (36.5)	104 (35.8)	0.0148	62 (35.8)	65 (37.6)	−0.0360
Not documented	32 (9.8)	38 (12.8)	−0.0955	41 (11.6)	34 (11.5)	0.0020	17 (9.8)	23 (13.3)	−0.1086
ECOG PS ^a^ *n* (%)									
0	126 (38.7)	62 (20.9)	0.3945	106 (29.7)	85 (29)	0.0158	43 (24.9)	41 (23.7)	0.0270
1	78 (23.9)	70 (23.6)	0.0065	87 (24.5)	70 (24.0)	0.0119	48 (27.7)	43 (24.9)	0.0657
2, 3, or 4	37 (11.3)	51 (17.2)	−0.1686	51 (14.3)	43 (14.6)	−0.0081	27 (15.6)	27 (15.6)	0.0000
Not documented	85 (26.1)	113 (38.2)	−0.2614	112 (31.4)	95 (32.4)	−0.0202	55 (31.8)	62 (35.8)	−0.0856
Bone-only metastasis ^a,b^ *n* (%)	0	0	0	0	0	0	0	0	0
Brain metastasis, n (%)	15 (4.6)	18 (6.1)	0.0659	14 (3.9)	23 (7.8)	0.1686	6 (3.5)	15 (8.7)	0.2192
Disease-free interval, years ^a,c^ *n* (%)									
De novo mBC	131 (40.2)	101 (34.1)	0.1257	130 (36.5)	104 (35.8)	0.0148	62 (35.8)	65 (37.6)	−0.0360
≤1	4 (1.2)	8 (2.7)	−0.1065	4 (1.2)	6 (2.2)	−0.0771	3 (1.7)	2 (1.2)	0.0485
>1 to 5	40 (12.3)	67 (22.6)	−0.2757	48 (13.4)	69 (23.6)	−0.2627	25 (14.5)	37 (21.4)	−0.1816
>5	151 (46.3)	120 (40.5)	0.1168	174 (48.9)	112 (38.5)	0.2110	83 (48.0)	69 (39.9)	0.1636
Not documented	0	0	0	0	0	0	0	0	0
NCI comorbidity index, mean (SD)	0.28 (0.51)	0.38 (0.53)	−0.1979	0.31 (0.59)	0.35 (0.50)	−0.0798	0.34 (0.61)	0.34 (0.50)	0.0097
Number of metastatic sites ^a,d^ *n* (%)									
1	45 (13.8)	64 (21.6)	−0.2059	62 (17.4)	51 (17.4)	0.0020	25 (14.5)	23 (13.3)	0.0334
2	105 (32.2)	101 (34.1)	−0.0406	121 (34.0)	100 (34.2)	−0.0030	64 (37.0)	60 (34.7)	0.0482
3	108 (33.1)	87 (29.4)	0.0807	107 (30.1)	95 (32.4)	−0.0509	54 (31.2)	59 (34.1)	−0.0617
4	40 (12.3)	23 (7.8)	0.1503	38 (10.8)	24 (8.0)	0.0943	17 (9.8)	17 (9.8)	0.0000
≥5	28 (8.6)	21 (7.1)	0.0556	27 (7.7)	23 (8.0)	−0.0122	13 (7.5)	14 (8.1)	−0.0216
Median follow-up duration (IQR), months	26.2 (24.8)	23.0 (30.3)		25.6 (25.4)	23.6 (30.2)		24.0 (24.5)	24.2 (31.2)	

^a^ Variable used in propensity score estimation. ^b^ Bone-only disease was defined as metastatic disease in the bone only. ^c^ Time from the initial diagnosis to the mBC diagnosis. ^d^ Multiple metastases at the same site were counted as one site (e.g., if a patient had three bone metastases in the spine, it was considered only one site). AI, aromatase inhibitor; ECOG PS, Eastern Cooperative Oncology Group performance status; IQR, interquartile range; mBC, metastatic breast cancer; NCI, National Cancer Institute; PSM, propensity score matching; SD, standard deviation; sIPTW, stabilized inverse probability of treatment weighting; Std Diff, standardized difference.

**Table 2:** Baseline and clinical characteristics of patients with liver metastasis.

Characteristic	Unadjusted			sIPTW			PSM		
	Palbociclib + AI (*n* = 211)	AI Alone (*n* = 165)	Std Diff	Palbociclib + AI (*n* = 237)	AI Alone (*n* = 179)	Std Diff	Palbociclib + AI (*n* = 85)	AI Alone (*n* = 85)	Std Diff
Age at mBC diagnosis, years ^a^									
Mean (SD)	62.9 (11.6)	70.0 (9.7)	−0.6704	65.8 (12.3)	66.6 (11.1)	−0.0686	67.0 (9.9)	68.6 (9.3)	−0.1713
Median (IQR)	63 (16)	71 (17)		67 (18)	67 (16)		68 (13)	69 (12)	
Female sex ^a^ *n* (%)	210 (99.5)	163 (98.8)	−0.0808	235 (99.4)	177 (99.3)	−0.0125	85 (100)	85 (100)	0.0000
Race ^a^ *n* (%)									
White	155 (73.5)	107 (64.8)	0.1873	168 (70.9)	127 (70.9)	0.0002	64 (75.3)	66 (77.6)	−0.0555
Black	10 (4.7)	17 (10.3)	−0.2121	12 (5.2)	11 (6.1)	−0.0374	5 (5.9)	3 (3.5)	0.1113
Other	46 (21.8)	41 (24.8)	−0.0721	56 (23.8)	41 (23.0)	0.0203	16 (18.8)	16 (18.8)	0.0000
Practice type			−0.0016			−0.0340			0.1718
Community	193 (91.5)	151 (91.5)		216 (91.3)	165 (92.2)		80 (94.1)	76 (89.4)	
Academic	18 (8.5)	14 (8.5)		21 (8.74)	14 (7.8)		5 (5.9)	9 (10.6)	
mBC disease stage at initial diagnosis, *n* (%)									
I	32 (15.2)	27 (16.4)	−0.0329	37 (15.6)	25 (14.2)	0.0406	9 (10.6)	11 (12.9)	−0.0731
II	60 (28.4)	32 (19.4)	0.2132	59 (25.0)	45 (25.2)	−0.0035	19 (22.4)	20 (23.5)	−0.0280
III	25 (11.8)	26 (15.8)	−0.1135	32 (13.7)	25 (14.0)	−0.0094	13 (15.3)	13 (15.3)	0.0000
IV	81 (38.4)	60 (36.4)	0.0419	87 (36.6)	69 (38.4)	−0.0372	36 (42.4)	38 (44.7)	−0.0475
Not documented	13 (6.2)	20 (12.1)	−0.2079	22 (9.1)	15 (8.3)	0.0296	8 (9.4)	3 (3.5)	0.2408
ECOG PS ^a^ *n* (%)									
0	86 (40.8)	41 (24.0)	0.3438	80 (33.8)	63 (35.1)	−0.0273	34 (40.0)	27 (31.8)	0.1723
1	49 (23.2)	27 (16.4)	0.1728	48 (20.1)	33 (18.7)	0.0346	14 (16.5)	17 (20.0)	−0.0915
2, 3, or 4	30 (14.2)	39 (23.6)	−0.2422	42 (17.9)	34 (19.1)	−0.0323	14 (16.5)	16 (18.8)	−0.0618
Not documented	46 (21.8)	58 (35.2)	−0.2991	67 (28.2)	48 (27.0)	0.0265	23 (27.1)	25 (29.4)	−0.0523
Bone-only metastasis ^a,b^ *n* (%)	0	0	0	0	0	0	0	0	0
Brain metastasis, *n* (%)	11 (5.2)	8 (4.8)	−0.0167	13 (5.4)	16 (8.8)	0.1324	5 (5.9)	5 (5.9)	0.0000
Disease-free interval, years ^a,c^ *n* (%)									
De novo mBC	81 (38.4)	60 (36.4)	0.0419	87 (36.6)	69 (38.4)	−0.0372	36 (42.4)	38 (44.7)	−0.0475
≤1	10 (4.7)	5 (3.0)	0.0885	9 (3.9)	5 (2.6)	0.0719	3 (3.5)	1 (1.2)	0.1557
>1 to 5	34 (16.1)	47 (28.5)	−0.3005	34 (14.6)	54 (30.5)	−0.3885	7 (8.2)	28 (32.9)	−0.6417
>5	86 (40.8)	52 (31.5)	0.1933	106 (45.0)	50 (28.1)	0.3564	39 (45.9)	18 (21.2)	0.5422
Not documented	0	1 (0.6)	−0.1104	0	1 (0.4)	−0.0935	0	0	0
NCI comorbidity index, mean (SD)	0.25 (0.40)	0.33 (0.50)	−0.1684	0.29 (0.46)	0.28 (0.48)	0.0200	0.29 (0.44)	0.27 (0.38)	0.0462
Number of metastatic sites ^a,d^ *n* (%)									
1	40 (19.0)	29 (17.6)	0.0358	44 (18.8)	31 (17.6)	0.0297	18 (21.2)	12 (14.1)	0.1860
2	69 (32.7)	63 (38.2)	−0.1148	84 (35.3)	65 (36.4)	−0.0223	29 (34.1)	31 (36.5)	−0.0493
3	52 (24.6)	41 (24.8)	−0.0047	54 (22.8)	42 (23.3)	−0.0131	19 (22.4)	24 (28.2)	−0.1356
4	26 (12.3)	16 (9.7)	0.0839	28 (11.7)	16 (9.0)	0.0900	9 (10.6)	10 (11.8)	−0.0373
≥5	24 (11.4)	16 (9.7)	0.0547	27 (11.4)	24 (13.7)	−0.0682	10 (11.8)	8 (9.4)	0.0765
Median follow-up duration (IQR), months	20.1 (21.4)	13.1 (20.4)		20.0 (21.4)	14.8 (19.1)		22.4 (21.5)	13.0 (21.2)	

^a^ Variable used in propensity score estimation. ^b^ Bone-only disease was defined as metastatic disease in the bone only. ^c^ Time from the initial diagnosis to the mBC diagnosis. ^d^ Multiple metastases at the same site were counted as one site (e.g., if a patient had three bone metastases in the spine, it was considered only one site). AI, aromatase inhibitor; ECOG PS, Eastern Cooperative Oncology Group performance status; IQR, interquartile range; mBC, metastatic breast cancer; NCI, National Cancer Institute; PSM, propensity score matching; SD, standard deviation; sIPTW, stabilized inverse probability of treatment weighting; Std Diff, standardized difference.

## Data Availability

The data that support the findings of this study were originated by Flatiron Health, Inc. These de-identified data may be made available on request and are subject to a license agreement with Flatiron Health. Interested researchers should contact this group to determine licensing terms and obtain the training, data dictionary, validation, and datasets. The Flatiron Health Analytic Database can be contacted at https://flatiron.com/contact/ (accessed on 30 October 2023). Interactive visualization of the data presented in this article is available at: https://realworld-data.dimensions.ai/p-reality-x (accessed on 30 October 2023).

## Supplementary Materials

The following supporting information can be downloaded at: https://www.mdpi.com/article/10.3390/cancers15215268/s1, Table S1: Baseline and clinical characteristics of patients with lung and/or liver metastases; Figure S1: OS for patients with lung and/or liver metastases in the unadjusted (A), sIPTW (B), and PSM (C) analyses. AI, aromatase inhibitor; CI, confidence interval; OS, overall survival; PAL, palbociclib; PSM, propensity score matching; sIPTW, stabilized inverse probability of treatment weighting; Figure S2: rwPFS for patients with lung and/or liver metastases in the unadjusted (A), sIPTW (B), and PSM (C) analyses. AI, aromatase inhibitor; CI, confidence interval; PAL, palbociclib; PSM, propensity score matching; rwPFS, real-world progression-free survival; sIPTW, stabilized inverse probability of treatment weighting.
