# Supplementary material for: Palbociclib Combined with an Aromatase Inhibitor in Patients with Breast Cancer with Lung or Liver Metastases in US Clinical Practice
Source: Cancers (Basel). 2023 Nov 2;15(21):5268. doi: 10.3390/cancers15215268 (PMC10649131; doi:10.3390/cancers15215268)

Supplementary Materials

Table S1. Baseline and clinical characteristics of patients with lung and/or liver metastases.

Characteristic	Unadjusted			sIPTW			PSM		
	**Palbociclib + AI (*n* = 480)**	**AI Alone (*n* = 411)**	**Std Diff**	**Palbociclib + AI (*n* = 524)**	**AI Alone (*n* = 428)**	**Std Diff**	**Palbociclib + AI (*n* = 256)**	**AI Alone (*n* = 256)**	**Std Diff**
Age at mBC diagnosis, years[a]									
Mean (SD)	65.5 (11.0)	70.6 (9.7)	−0.4959	67.6 (11.5)	68.2 (10.6)	−0.0590	68.1 (10.7)	69.4 (9.4)	−0.1335
Median (IQR)	66 (14)	71 (16)		69 (17)	69 (16)		69 (16)	70 (16)	
Female sex[a] *n* (%)	476 (99.2)	403 (98.1)	−0.0952	515 (98.3)	422 (98.6)	0.0223	253 (98.8)	254 (99.2)	0.0397
Race[a] *n* (%)									
White	331 (69.0)	256 (62.3)	0.1408	350 (66.8)	286 (66.9)	−0.0023	164 (64.1)	180 (70.3)	−0.1334
Black	31 (6.5)	39 (9.5)	−0.1121	39 (7.4)	31 (7.3)	0.0016	18 (7.0)	13 (5.1)	0.0820
Other	118 (24.6)	116 (28.2)	−0.0827	135 (25.8)	110 (25.8)	0.0016	74 (28.9)	63 (24.6)	0.0972
Practice type									
Community	441 (91.9)	376 (91.5)	0.0142	481 (91.8)	392 (91.6)	0.0059	227 (88.7)	233 (91.0)	−0.0776
Academic	39 (8.1)	35 (8.5)		43 (8.2)	36 (8.4)		29 (11.3)	23 (9.0)	
mBC disease stage at initial diagnosis, *n* (%)									
I	63 (13.1)	58 (14.1)	−0.0288	71 (13.6)	58 (13.5)	0.0033	36 (14.1)	40 (15.6)	−0.0440
II	135 (28.1)	92 (22.4)	0.1324	135 (25.7)	113 (26.3)	−0.0150	76 (29.7)	70 (27.3)	0.0519
III	53 (11.0)	64 (15.6)	−0.1337	68 (12.9)	57 (13.3)	−0.0110	28 (10.9)	31 (12.1)	−0.0367
IV	185 (38.5)	145 (35.3)	0.0676	190 (36.2)	153 (35.8)	0.0089	87 (34.0)	87 (34.0)	0.0000
Not documented	44 (9.2)	52 (12.7)	−0.1120	61 (11.6)	47 (11.1)	0.0153	29 (11.3)	28 (10.9)	0.0124
ECOG PS[a] *n* (%)									
0	189 (39.4)	94 (22.9)	0.3623	166 (31.6)	134 (31.3)	0.0068	72 (28.1)	76 (29.7)	−0.0345
1	112 (23.3)	87 (21.2)	0.0521	119 (22.6)	93 (21.8)	0.0210	54 (21.1)	57 (22.3)	−0.0284
2, 3, or 4	55 (11.5)	78 (19.0)	−0.2105	80 (15.2)	66 (15.3)	−0.0045	38 (14.8)	38 (14.8)	0.0000
Not documented	124 (25.8)	152 (37.0)	−0.2420	160 (30.6)	135 (31.6)	−0.0223	92 (35.9)	85 (33.2)	0.0575
Bone-only metastasis[a,b] *n* (%)	0	0	0	0	0	0	0	0	0
Brain metastasis, *n* (%)	22 (4.6)	19 (4.6)	0.0019	23 (4.3)	26 (6.1)	0.0801	12 (4.7)	16 (6.3)	0.0688
Disease-free interval, years[a,c] *n* (%)									
De novo mBC	185 (38.5)	145 (35.3)	0.0676	190 (36.2)	153 (35.8)	0.0089	87 (34.0)	87 (34.0)	0.0000
≤1	12 (2.5)	12 (2.9)	−0.0259	12 (2.2)	11 (2.5)	−0.0214	5 (2.0)	4 (1.6)	0.0297
>1–5	66 (13.8)	97 (23.6)	−0.2548	73 (14.0)	108 (25.3)	−0.2863	39 (15.2)	67 (26.2)	−0.2724
>5	217 (45.2)	156 (38.0)	0.1475	249 (47.6)	155 (36.2)	0.2315	125 (48.8)	98 (38.3)	0.2139
Not documented	0	1 (0.2)	−0.0698	0	1 (0.2)	−0.0622	0	0	0
NCI comorbidity index, mean (SD)	0.27 (0.47)	0.36 (0.52)	−0.2001	0.30 (0.54)	0.33 (0.49)	−0.0490	0.28 (0.48)	0.33 (0.48)	−0.1160
Number of metastatic sites[a,d] *n* (%)									
1	85 (17.7)	93 (22.6)	−0.1228	104 (19.9)	84 (19.7)	0.0054	45 (17.6)	46 (18.0)	−0.0102
2	169 (35.2)	160 (38.9)	−0.0771	197 (37.6)	163 (38.0)	−0.0074	101 (39.5)	98 (38.3)	0.0240
3	139 (29.0)	107 (26.0)	0.0655	136 (26.0)	122 (28.6)	−0.0592	70 (27.3)	73 (28.5)	−0.0261
4	53 (11.0)	28 (6.8)	0.1487	51 (9.8)	30 (7.0)	0.1022	30 (11.7)	23 (9.0)	0.0899
≥5	34 (7.1)	23 (5.6)	0.0611	35 (6.7)	29 (6.8)	−0.0020	10 (3.9)	16 (6.3)	−0.1069
Median follow-up duration (IQR), months	24.5 (23.7)	20.5 (28.2)		24.2 (24.0)	21.4 (27.2)		24.0 (23.9)	21.2 (26.8)	

[a]Variable used in propensity score estimation. [b]Bone-only disease was defined as metastatic disease in the bone only. [c]Time from the initial diagnosis to mBC diagnosis. [d]Multiple metastases at the same site were counted as one site (eg, if a patient had three bone metastases in the spine, it was considered only one site).

AI, aromatase inhibitor; ECOG PS, Eastern Cooperative Oncology Group performance status; IQR, interquartile range; mBC, metastatic breast cancer; NCI, National Cancer Institute; PSM, propensity score matching; SD, standard deviation; sIPTW, stabilized inverse probability of treatment weighting; Std Diff, standardized difference.

Figure S1. OS for patients with lung and/or liver metastases in the unadjusted (A), sIPTW (B), and PSM (C) analyses. AI, aromatase inhibitor; CI, confidence interval; OS, overall survival; PAL, palbociclib; PSM, propensity score matching; sIPTW, stabilized inverse probability of treatment weighting.

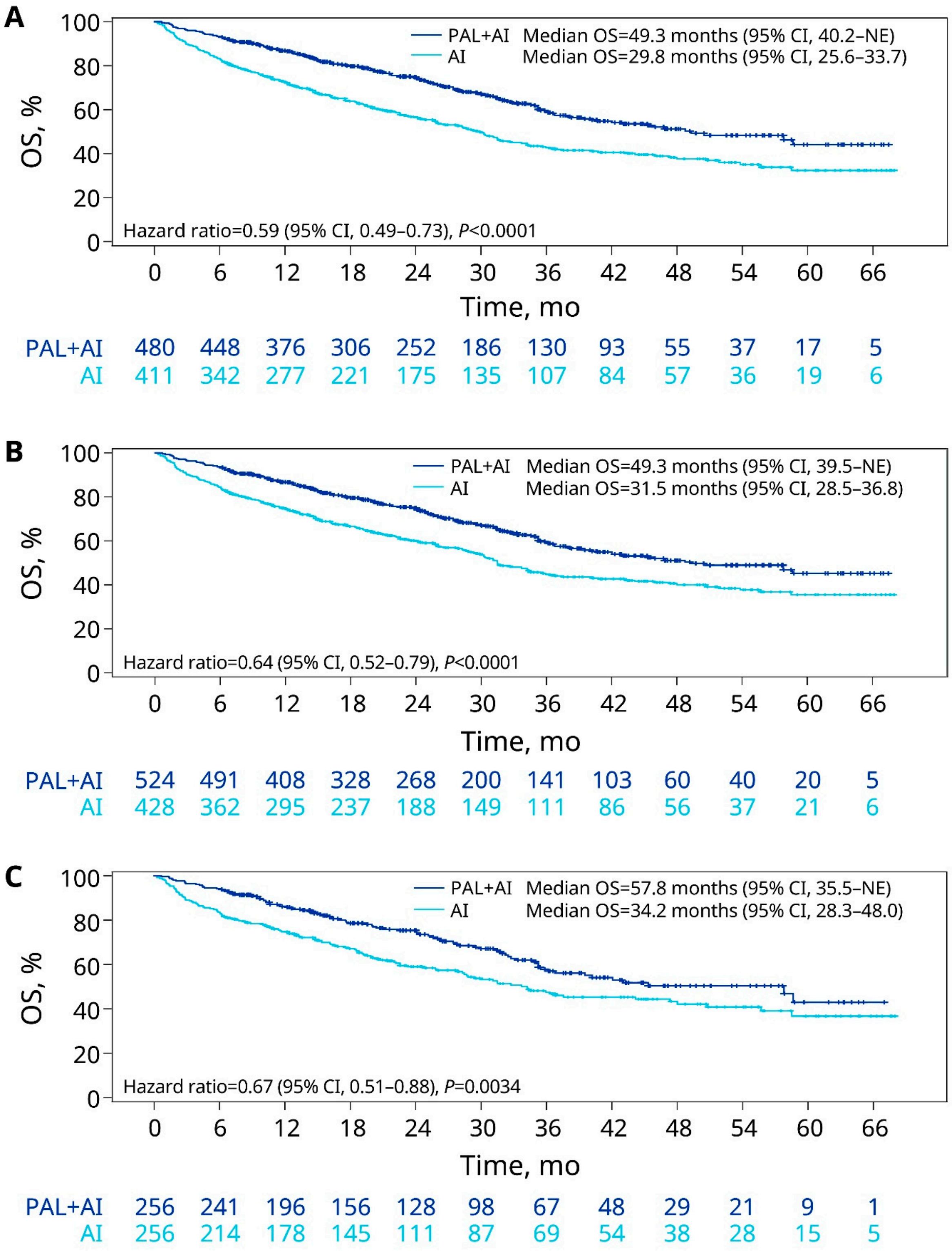

Figure S2. rwPFS for patients with lung and/or liver metastases in the unadjusted (A), sIPTW (B), and PSM (C) analyses. AI, aromatase inhibitor; CI, confidence interval; PAL, palbociclib; PSM, propensity score matching; rwPFS, real-world progression-free survival; sIPTW, stabilized inverse probability of treatment weighting.

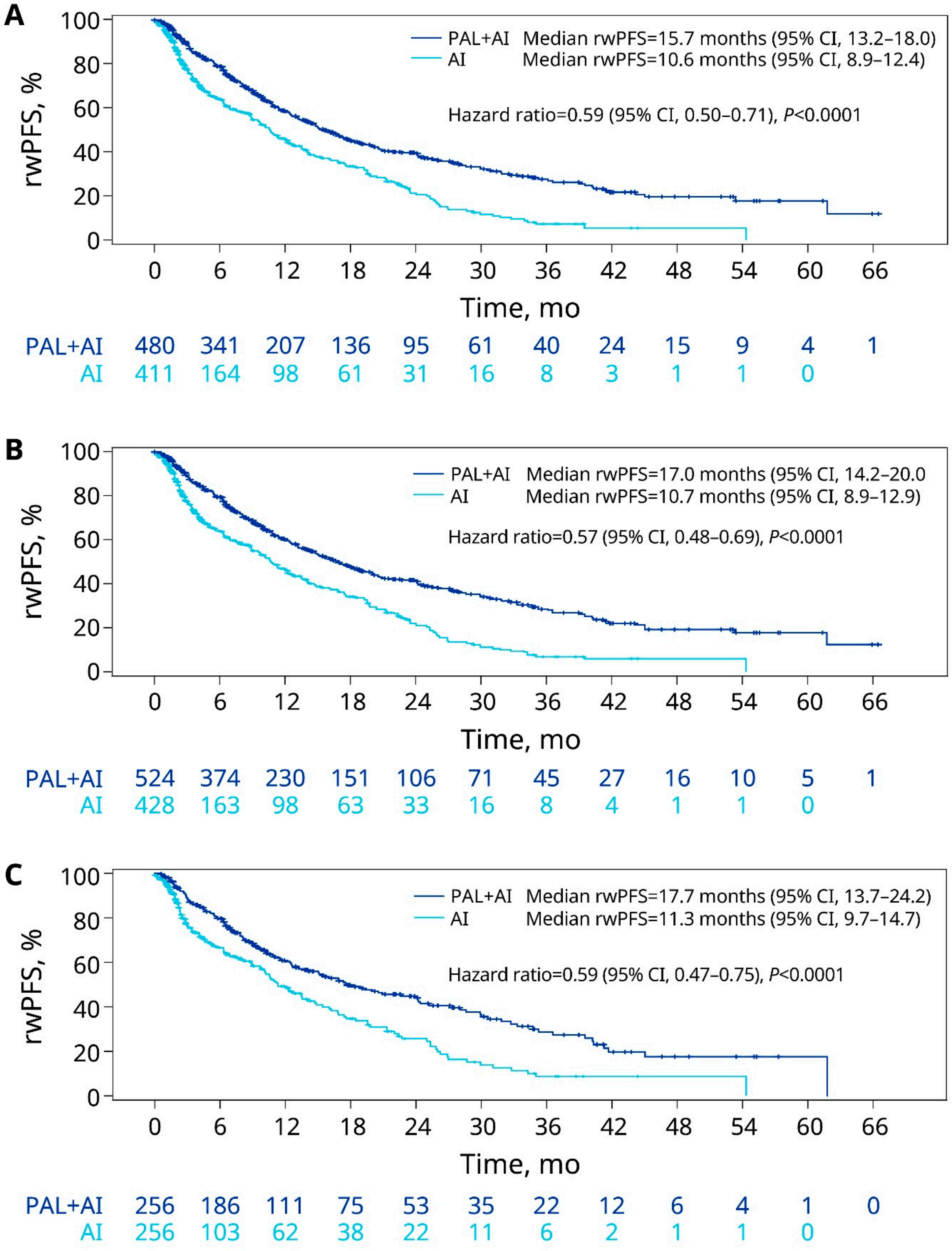

Supplement: Supplementary file 1 [file cancers-15-05268-s001.zip › cancers-2674513-supplementary.pdf]
